# The Role of Anticoagulation Clinics in the Era of New Oral Anticoagulants

**DOI:** 10.1155/2012/835356

**Published:** 2012-10-14

**Authors:** Sophie Testa, Oriana Paoletti, Anke Zimmermann, Laura Bassi, Silvia Zambelli, Emilia Cancellieri

**Affiliations:** Haemostasis and Thrombosis Center, A.O. Istituti Ospitalieri, Cremona, Italy

## Abstract

Anticoagulation Clinics (ACs) are services specialized in management of patients on anticoagulant treatment. At present, ACs manage patients chiefly on antivitamin K antagonists (AVKs), but patient population has already changed in the last few years, because of an increase of treatments with other anticoagulant drugs, which require different management systems. The strong increase in the number of patients at AC, mainly on long-term treatment, has determined the development of web management, through telemedicine systems, improving the quality of life and maintaining the same clinical quality levels. New oral anticoagulants (NOAs) have shown to be as effective as AVK antagonists in stroke prevention in atrial fibrillation and for treatment of venous thromboembolism in addition to VTE prophylaxis in orthopaedic surgery, when administered at a fixed dose, but patient adherence and compliance are crucial for good quality treatment. At present, lacking data from the real world, an oversimplification of treatment with NOAs could cause unjustified risks for patients and also a possible future underuse of good drugs. For these reasons the vigilance must be high and ACs can have a crucial role in defining which is the best management for NOA patients and how to do it, as it happened for AVKs.

## 1. Introduction

Anticoagulation Clinics (ACs) are services specialized in management of patients on anticoagulant treatment. They can be established as a section of a Thrombosis Center-multifunctional services with clinical and laboratory expertees, wich provide diagnosis, treatment and prevention of thromboembolic diseases- or act as independent services.

At present, ACs manage patients chiefly on antivitamin K antagonists (AVKs), but patient population has already changed in the last few years, because of an increase of treatments with other anticoagulant drugs, such as low molecular weight heparin (LMWH) or pentasaccharides, which require different management systems. 

AVKs, that until few months ago were the only oral available anticoagulant agents, are life-saving therapies that can effectively prevent cardioembolic strokes related to atrial fibrillation and heart valve replacements and treat venous thromboembolism. Because of their characteristics AVKs need a strict laboratory and clinical control to ensure efficacy and safety [1]. In fact major bleeding and thrombotic complications are mainly related to coagulation levels out of ranges, age, comorbidities, comedications, and poor therapeutic control [2, 3]. Therefore effectiveness and safety of AVKs increase when a good control of anticoagulation level is guaranteed. For this reason, a correct use of AVK requires a careful clinical and laboratory monitoring, as well as specific competences for managing complications and/or emergencies [1].

## 2. Anticoagulation Clinic 

For AVKs we can consider 4 different management models: (1) routine medical care (RMC), (2) AC, (3) patient self testing (PST), and (4) patient self-management (PSM). The four models for AVK control are variously developed in different countries. Several studies have shown that anticoagulation management is crucial to ensure quality of treatment and, among different types of management, and Anticoagulation Clinic represent the best management model (Table 1) [4–13]. 

Aims of ACs are the following:

- determining the appropriate clinical indications for anticoagulant treatment;
- determining the laboratory tests necessary to pharmacological monitoring;
- prescribing the anticoagulation regimen based on the results of the laboratory tests;
- defining the time intervals for regular anticoagulation controls;
- assessing the potential pharmacological interactions;
- taking care of patients undergoing surgical interventions;
- carrying out educational programs for patients and healthcare providers.

The increase of AVK population in the last thirty years is related to many factors, including the good organization of ACs and the standardisation of laboratory methods, which has allowed to perform clinical trials in order to demonstrate AVK efficacy and safety [4]. These studies have supplied a better knowledge of therapeutic indications, optimal anticoagulation levels (therapeutic ranges), risk of haemorrhagic and thrombotic complications, pharmacological interferences, and the importance of a specialistic management [6]. 

## 3. Anticoagulation Clinic ****and Telemedicine Development

The strong increase in the number of patients at AC, mainly on long-term treatment, has determined the development of web management, through telemedicine systems [14, 15], in some areas.

Telemedicine systems for AVK have been developed during the last few years with the aim to decentralize in the health territorial care units the activity of ACs, improving the quality of life of patients living far from the AC site and maintaining the same clinical quality levels.

Until now, in daily clinical practice AVKs are still underused because they are often considered a therapy that can be hardly managed, laborious, and potentially dangerous for the patient, therefore an integration between hospital services and health care units can facilitate access to life-saving therapies.

Telemedicine systems for anticoagulated patients should be structured through net supported programs to collect and elaborate clinical data, connected among hospital divisions, health care peripheral districts, and patients. All information, including clinical data, laboratory controls, alerts, prescriptions, and recommendations have to be available in real time through a bidirectional connection [15, 16]. 

Telemedicine applied to anticoagulated patients offers several advantages, both for organization as well as for improving communications. Decentralize clinical activity through a telemedicine system gives the opportunity to redistribute patient population into different health care areas, whose medical services could be otherwise underused. It also can help accessibility to health care services for an increased number of patients, empowering management possibilities. At the same time the web gives the opportunity to export quality procedures and competences outside of specialized centres, increasing communications, not only between the physician and patient, but also between different clinical specialties.

Telemedicine organizations are opportunities to improve health care and patient's quality of life through a capillary distribution of medical assistance, supporting physicians in the application of good clinical practice in anticoagulation management. 

As anticoagulant drugs are changing through the introduction of new antithrombotic drugs, telemedicine could help their management. 

New-generation antithrombotic drugs will need different types of management compared with AVKs, with a less frequent clinical and laboratory control. However, it will be necessary to follow up and record patient's data to guarantee the strict quality control on adherence, compliance, incidence of intercurrent diseases, adverse events, and complications. 

The advantage of a web organization can be summarized as following:

- accessibility for a higher number of patients to a high quality management system;
- integration among different specialists and health structures;
- continuing medical record update;
- possibility to manage patients on different antithrombotic drugs.

The web gives the possibility to increase quickly connections by modulating the management in relation to different molecules, assuring clinical control for all patients. 

## 4. New Oral Anticoagulant Drugs

While AVK antagonists are indirect agents that act blocking liver synthesis of vitamin K coagulation factors, NOAs are synthetic molecules that inhibit directly a single target coagulation factor (Table 2) [17, 18]. New agents are already available in some countries: dabigatran, a direct anti-FIIa, and rivaroxaban, an anti-FXa. Apixaban and Edoxaban, two other anti-FXa, are in advanced evaluation state (see details in previous articles). 

Potential advantages as compared to VKA are:

- rapid onset of action without need for bridging therapy,
- predictable anticoagulant effect without need for dose-adjustment laboratory testing, and
- low food/drug interactions without need for restrictions.

Each agent has specific pharmacodynamic and pharmacokinetic characteristics (half-life, bioavailability, route of elimination, posology, frequency of administration, and potential drug interactions) that should be well known by prescribers (Table 2). 

NOAs have shown to be as effective as AVK antagonists in stroke prevention in atrial fibrillation and for treatment of venous thromboembolism [19–22] in addition to VTE prophylaxis in orthopaedic surgery [23]. Studies published until now show similar safety, but a reduction in major cerebral bleeding for dabigatran, rivaroxaban, and apixaban [19–23].

Even if NOAs proved to be effective and safe when administered at a fixed dose, laboratory tests could be useful in clinical practice for measuring the anticoagulant effect in specific conditions such as: bleeding and thrombotic complications, surgery or invasive procedures, renal impairment, liver disease, and potential overdosages [24]. 

Several studies have been recently published showing different coagulation test sensitivity for each molecule [24–27]. Currently few data are available to recommend the most appropriate test for each drug.

## 5. Patient Management on NOA

Patient adherence and compliance are crucial for NOA good quality treatment, but not for AVK patients, because the systematic laboratory controls improve the adherence to therapy. Usually, patients taking chronic drugs have poor medication-taking behavior [28]. Literature shows that even after an emergency, such as an acute coronary syndrome, the continuous use of life-saving drugs after 6–12 months drops to 71% for aspirin, 46% for beta-blockers, and 44% for statins, with the adherence to all the three medications being only 21% [29]. Estimates of nonadherence in the elderly (defined as those aged ≥65 years) varies from 40% to 75%, especially in chronic asymptomatic conditions like hypertension or as could be atrial fibrillation. 

It would be desirable that clinical followup is organized. Among prescribing physician, general practitioner, other specialties, and Anticoagulation Clinic the last one has more competences, as previously described. 

Alternatively, other less effective measures to improve compliance are:

- phone call by nurses,
- log book to be filled by patients,
- broad education at time of prescription, and
- specific education with pamphlets and questionnaires.

At present, laboratory monitoring is not recommended in patients taking NOAs because phase III studies have shown efficacy and safety in administration at fixed dosage [19–23]. However particular clinical conditions in real world population will require and need laboratory control coagulation test to evaluate haemostatic balance.

For example:

- before surgery to assess haemostatic system and consequently the safety of invasive procedures,
- in patients with bleeding/thromboembolic complications to evaluate a correlation between events and over-/undertreatment,
- in elderly patients, who physiologically have renal impairment, or in underweight and obese patients to evaluate drug accumulation,
- in renal/liver disease, and
- potential pharmacological interactions.

## 6. The Anticoagulation Clinic in the Future

If it is true that each type of anticoagulant drug has specific pharmacodynamic and pharmacokinetic characteristics, then we must consider different types of clinical/laboratory management in relation to their proper characteristics.

For all of them (AVK and NOA) we have to consider the following items (Table 3):

1. determine the appropriate clinical indications for anticoagulant treatment;
2. choose the best drug in relation to clinical patient profile;
3. evaluate any potential pharmacological interference, in particular in the elderly;
4. control drug adherence, that for AVKs is guaranteed through laboratory test;
5. define a follow-up program;
6. define educational programs to increase adherence;
7. manage patients undergoing surgical interventions or invasive procedures;
8. manage patients with bleeding and thrombotic complications, during anticoagulant treatment;
9. manage patients during intercurrent diseases;
10. choose the best laboratory test for each molecule to support clinical and therapeutical activities.

As previously discussed compliance of patients that require long-term therapy is a crucial problem and represents an important factor of variability of both quality of treatment and patient management. Lacking evidences for NOAs laboratory monitoring, it is required a strict control of compliance and adherence to avoid major complications due to under-/overdosages [30–35].

Educational courses for patients (indications for treatment, risk and benefit, problem in drug administration, dietary behaviour, interactions with other drugs, and intercurrent disease) and for health territorial care units can improve patient's compliance [36].

ACs are specialized services, well experienced on anticoagulant treatment and they are able to support patients on critical clinical conditions, mainly because of their knowledge of coagulation mechanisms. This means that they can easily understand complication etiopathogenesis and quickly provide specific therapeutic interventions in emergency conditions. As an example we can consider over dosages on AVK treatment and the correct use of prothrombin concentrates or preparation of patients on anticoagulant treatment for surgical intervention, through assessing the normalization of haemostatic parameters.

An advantage of ACs is also the complete recording of clinical and laboratory data, always available, necessary for clinical surveillance and pharmacovigilance evaluation.

Taking into account advantages provided by an AC an integration between hospital services and primary care units is desirable to standardize quality of treatment on anticoagulation. Telemedicine systems offer the opportunity to empower the integration among different heath care providers, ensuring standard level of care among specialists, capillary diffusion of anticoagulant treatments, homogeneous data collection, continuing education, real time evaluation of major complications and comparison among different drugs, and improving knowledge and information on NOAs.

This integration promotes communication among different professional activities (nurses, physicians) and it can help to control compliance and adherence of anticoagulated patients, assuring an optimal standard of care. In particular, in the initial period of the use of NOAs it is necessary a strict patient control to avoid errors in their clinical use, regarding correct indication, posology, adherence and compliance, patient management in special conditions like surgery, invasive procedures, or intercurrent diseases. At present, lacking data from the real world, an oversimplification of treatment with NOAs could cause unjustified risks for patients and also a possible future under use of good drugs.

For all these reasons, in these first years of introduction of NOAs, vigilance must be high and ACs can have a crucial role in defining which is the best management for NOA patients and how to do it, as it happened for AVKs.

## Figures and Tables

**Table 1:** Principal characteristics of the four management models.

	RMC	AC	PSM	PST
Clinical quality	+	++++	++++	++++
TTR	+	+++	+++	++++
Accessibility	++++	++	+	++
Costs	+	++	+++	++++

**Table 2:** Principal characteristics of new oral anticoagulants ([18, 19], modified).

	Dabigatran	Rivaroxaban	Apixaban	Edoxaban
Target	IIa	Xa	Xa	Xa
Prodrug	Yes	No	No	No
Hours to*C*_max⁡_	2	2–4	1–3	1.5
Bioavailability	7%	80%	66%	50%
Half-life (hours)	12–14	9–13	8–15	6–11
CYP metabolism	No	Yes	Yes	Yes
Efflux transporter P-gp	Yes	Yes	Yes	Yes
Renal elimination	80%	66% (33% cleared unchanged)	25%	35%
Dosing	Bid	Od	Bid	Od

**Table 3:** Patients management: comparison between NOA and AVK clinical actions.

	AVK	NOA
Visit (anamnesis, physical examination)	Yes	Yes
Prescription (clinical indication, posology)	Yes	Yes
Information/education	Yes	Yes
Frequent lab coagulation monitoring	Yes	No
Renal function control	No	Yes
Dosing adjustment	Yes	No
Adherence/compliance control	No (routinely evaluated)	Yes
Management in special situations (surgery/complications)	Yes	Yes
Clinical periodical control	No (routinely evaluated)	Yes
Lab testing to support clinical and therapeutical intervention in specific condition	Yes	Yes (under evaluation)
